# Microstructure, Tribological, and Corrosion Behavior of HVOF-Sprayed (Cr_3_C_2_-NiCr+Ni) Coatings on Ductile Cast Iron

**DOI:** 10.3390/ma18081856

**Published:** 2025-04-18

**Authors:** Marzanna Ksiazek, Lukasz Boron

**Affiliations:** 1 Department of Non-Ferrous Metals, AGH University of Krakow, al. A. Mickiewicza 30, 30-059 Krakow, Poland; 2 Lukasiewicz Research Network-Krakow Institute of Technology, 73 Zakopianska Str., 30-418 Krakow, Poland; lukasz.boron@kit.lukasiewicz.gov.pl

**Keywords:** Cr_3_C_2_-NiCr coating, HVOF spraying, microstructure, instrumented indentation, corrosion test

## Abstract

The HVOF (High Velocity Oxy-Fuel) thermal spraying method is widely used in surface engineering to produce coatings with high hardness, low porosity, and excellent crack resistance. Composite coatings with chromium carbide (Cr_3_C_2_) in a nickel–chromium (NiCr) matrix are commonly applied in demanding environments, such as the energy and transport sectors. This study compares the microstructure, mechanical, tribological, and corrosion properties of two coatings—Cr_3_C_2_-25(Ni20Cr)-10(Ni) and Cr_3_C_2_-25(Ni20Cr)—deposited on ductile cast iron using HVOF. The addition of 10 wt.% Ni enhances coating integrity, mechanical performance, and environmental resistance by improving ductility, reducing residual stress, enhancing wettability, and balancing hardness with improved crack, wear, and corrosion resistance. Microstructure analysis via LM (Light Microscopy) and SEM (Scanning Electron Microscopy), along with chemical and phase characterization using EDS (Energy Dispersive X-ray Spectroscopy) and XRD (X-ray Diffraction), revealed that the Ni-enriched Cr_3_C_2_-25(Ni20Cr)-10(Ni) coating exhibited a denser structure, lower porosity, and high hardness. Its microstructure consists of large, partially melted Ni particles and fine Cr_3_C_2_ and Cr_7_C_3_ carbides embedded in the NiCr matrix, some at submicron scales. Performance tests, including indentation (H_IT_, E_IT_, K_IC_), scratch, and corrosion resistance assessments, confirmed that Ni addition improves crack resistance, wear durability, and corrosion protection. Consequently, these coatings demonstrate superior operational durability, making them more effective in challenging environments.

## 1. Introduction

The HVOF (High Velocity Oxygen Fuel) technology is one of the most advanced methods of producing protective coatings, combining high process efficiency with excellent properties of the obtained materials [1,2,3]. When this method is used, powder particles are deposited in a partially melted state at speeds of up to approximately 900 m/s, which makes it possible to obtain dense, fine-grained coatings with high hardness and excellent adhesion to the substrate. An additional advantage of HVOF is the minimal impact of the process on the substrate microstructure and the possibility of obtaining coatings of considerable thickness. One of the key advantages of HVOF technology is its ability to produce ceramic and cermet coatings with various chemical and phase compositions [4,5,6]. This makes it a competitive alternative to other thermal spraying methods, especially in applications requiring high resistance to abrasion, high temperature and corrosion [7,8]. Moreover, this technique is distinguished by the possibility of applying coatings in situ, for example in boiler systems, which gives it an advantage over CVD or PVD methods [9]. Coatings composed of chromium carbide (Cr_3_C_2_) embedded in a nickel–chromium (NiCr) matrix, applied using the HVOF technique, are extensively utilized across the energy, transportation, petrochemical, and marine sectors [10,11,12]. They are distinguished by high hardness, structural stability at elevated temperatures (up to 870 °C), and resistance to abrasion, erosion, and corrosion [13,14]. The characteristic microstructure of these coatings, characterized by low porosity (a few percent) and uniform distribution of fine-grained Cr_3_C_2_ particles in the NiCr matrix, is crucial for their excellent mechanical and tribological properties. The nickel–chromium matrix provides protection against corrosion, while chromium carbide is responsible for resistance to mechanical wear [15,16]. Partial decarburization of Cr_3_C_2_ occurs during the HVOF process, which leads to the formation of secondary carbide phases such as Cr_7_C_3_ and Cr_23_C_6_. The presence of these phases increases the surface smoothness and abrasion resistance, but may also limit the fracture toughness, especially in the case of smaller carbide particles [17,18,19,20]. The introduction of nano-structured Cr_3_C_2_-NiCr coatings enables further improvement of tribological properties due to increased content of grain boundaries in the microstructure, which translates into higher hardness and strength. However, the larger surface area to grain volume ratio in nano-structured powders promotes more intensive distribution of the Cr_3_C_2_ phase, which may negatively affect fracture toughness. In recent years, research has focused on modifying the composition of ceramic powders by introducing metal and nonmetal admixtures, such as Cr, Co, B, or Ni, as well as utilizing intermediate layers and additional coating processes. These activities help to limit the decarburization of Cr_3_C_2_, increasing the abrasion resistance and at the same time minimizing the susceptibility to brittle fracture [21,22,23,24,25,26,27]. Among these, the addition of Ni has gained particular attention due to its ability to enhance coating integrity, improve wettability, and reduce residual stresses. Ni also promotes a denser microstructure with lower porosity, leading to improved crack resistance, wear performance, and corrosion protection. These advancements contribute to further improving the performance properties and service life of carbide-based coatings. Thanks to these solutions, it is possible to further improve the performance properties of coatings. The corrosion properties of coatings in aggressive environments, such as NaCl, H_2_SO_4_ solutions, or alkaline environments, have been widely studied [28,29,30]. A key factor influencing their electrochemical behavior is the microstructure, which includes the presence of pores, micro-cracks and macro-cracks that favour electrolyte penetration. These phenomena can lead to the initiation of galvanic corrosion, degradation of the coating, and weakening of the substrate.

The aim of this study is to investigate the correlation between the microstructure, mechanical and tribological properties, and corrosion resistance of (Cr_3_C_2_-NiCr+Ni) composite coatings produced by the HVOF method on a ductile cast iron substrate. In response to the growing demand for innovative coatings with improved tribological properties, the analysis of their behavior in the context of wear and corrosion processes is a significant contribution to the development of advanced surface protection technologies.

## 2. Materials and Methods

### 2.1. Materials

**Figure 1:**
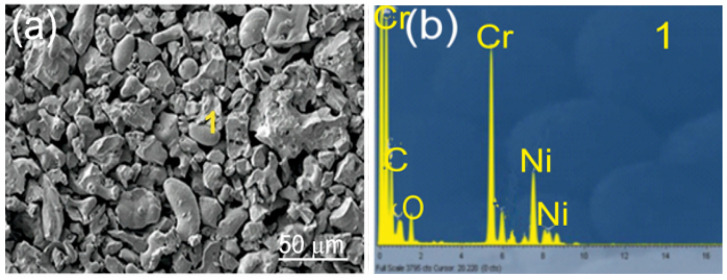
SEM/EDS of Cr3C2-25(Ni@0Cr) coating powder: (**a**) SEM micrograph; (**b**) EDS spectrum taken from the marked point 1.

The substrates consisted of EN-GJS-500-7 ductile iron plates with a chemical composition listed in Table 1 and mechanical properties detailed in Table 2. The samples measured 100 × 15 × 5 mm^3^. Before the spraying process, the substrate surfaces were sandblasted using loose corundum with a 20-mesh granulation to enhance mechanical adhesion. The resulting surface roughness parameter, Ra, was 30 µm. Coatings: Cr_3_C_2_-NiCr and (Cr_3_C_2_-NiCr+Ni) were applied by supersonic flame spraying from carbide powder with the following composition: Cr_3_C_2_-25NiCr (75 wt.% Cr_3_C_2_-25 wt.% NiCr) with a grain size of −45 + 15 µm (Diamalloy 3004 Salzer-Metco, Pfattikon, Switzerland) on a ductile cast iron substrate. Figure 1 shows an example of the carbide powder morphology (75%Cr_3_C_2_-25%NiCr) intended for the spraying process. The chemical composition of the powder at Point 1 is shown in Figure 1b. The (Cr_3_C_2_-NiCr+Ni) coating was obtained by introducing into the carbide powder 10 wt.% of Ni particles with nominal particle size distribution of −75 + 45 µm (Metco 56CNS, Oerlicon Metco, Westbury, NY, USA). The powders were pre-mixed before being introduced into the spraying device. The volume composition of the powder mixture used to create the composite coating was as follows: 67.5 wt.% Cr_3_C_2_-22.5 wt.% NiCr-10 wt.% Ni.

### 2.2. Coating Deposition

A supersonic spray system, HV-50 HVOF System at Plasma System SA company (Siemianowice, Silesia, Poland) was used to spray the coatings. The system uses a mixture of aviation kerosene and oxygen as fuel for the spraying process. The coating application parameters are listed in Table 3. The average thickness of the applied coating was 250 μm.

### 2.3. Microstructure Characterization

For testing of the microstructure and chemical composition of the coating/substrate- type system, a light microscope (LM) Axio Observer Zm1 by Zeiss (LM, Jena, Germany) and a Scios DualBeam FEI scanning electron microscope (SEM), (Thermo Fisher Scientific, Waltham, MA, USA) were used. Phase composition analyses were carried out on a diffractometer X’Pert Pro Panalytical (Malvern Panalytical Ltd., Cambridge, UK) in the angular range of 20–90° with CuK radiation. After such measurements, the obtained spectra were subjected to preliminary numerical processing using “EVA” software (Difrac. Eva V4), which consisted in cutting off the background and reducing noise using the Fourier transform. Phase identification was carried out with the help of ICDD database. Based on Rietveld analysis of XRD data with the use of GSAS/EXPGUI set of software (https://subversion.xray.aps.anl.gov/EXPGUI, accessed on 16 April 2025) phase composition was derived. The average crystallite size was calculated from the Scherrer formula upon taking into account the instrumental broadening. The composition of the surface layer of as-coated specimens was detected by X-ray diffraction (XRD) (Rigaku Co., Tokyo, Japan) with CuKα (λ = 1.54056 Å) in small steps of 2Ѳ = 0.02°. The average crystal size of the phases in the coated layer was calculated using Debye–Scherrer equation [31]. The carbide coating porosity was measured using X-ray computed tomography on a Pheonix Nanotom X-ray nanotomograph (GE Sensing & Inspection Technologies GmbH, Frankfurt, Germany), equipped with AxioVision image analysis software (4.8.2.0). The tests were conducted for ten areas in the coating. The analysis of the coating surface topography and determination of surface roughness parameters: R_a_ (arithmetical mean deviation from the mean line) and R_z_ (the maximum roughness height based on the measured 10 highest profile peaks) were measured on a LEXT OLS 4100 confocal laser microscope from OLYMPUS Corporation (Tokyo, Japan). The surface roughness parameters of the coatings were determined for three measurement lines for each type of coating. Three-dimensional images and their analysis allowed for precise understanding of the geometric structure of the surfaces tested.

### 2.4. Mechanical and Tribological Properties

**Figure 2:**
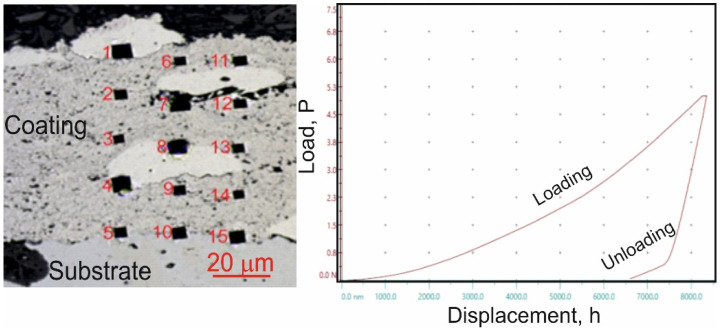
Measurement of microhardness (H_IT_) using a matrix distribution on the cross-section of the coating and the loading-depth curve of indentation in the matrix of the (Cr_3_C_2_-NiCr+Ni)/ductile cast iron system.

Micro-mechanical property tests, including microhardness, Young’s modulus, and fracture toughness measurements, were conducted using a Micro Combi Tester (CSM Instruments, Peseux, Switzerland), a multifunctional measurement platform. The values of H_IT_, E_IT_, and K_IC_ were determined through sample indentation on coating/substrate cross-sections using a Vickers diamond indenter. During testing, the forces and penetration depths of the indenter were continuously recorded throughout the loading and unloading cycle. The maximum applied load for hardness and Young’s modulus measurements was 1 N, with a loading and unloading rate of 2 N/min, a 10 s hold at peak load and a contact force of 0.03 N. For each cycle, the indenter load versus penetration depth was analyzed. The evaluation of micro-mechanical properties followed the Oliver and Pharr method, which was used to calculate microhardness (H_IT_) and Young’s modulus (E_IT_) from the indentation curve (Figure 2). Microhardness measurements were performed using a matrix layout of 15 test points across the coating cross-section (Figure 2). The precise measurement positions were determined using the “Visual Advanced Matrix” module, aided by an integrated light microscope.

The fracture toughness was determined by determining the K_IC_ parameter, i.e., the critical value of the stress concentration ratio by direct measurement of the length of cracks that formed in the corners as a result of the penetration of the Vickers indenter under a given load: 5, 10, 15, and 20 N (the load and unload speed was 40 N/min, the maximum load holding time was 10 s, and the contact force was 0.03 N). For this purpose, the crack lengths and the lengths of the indentation diagonals were determined using the integrated light microscope (Table 7 and Table 8). Three indentations were made in each coating/substrate-type sample at a given load. After determining the total length of the cracks, the type of cracks was identified, taking into account the length ratio l/a. If the l/a ratio is >1.5, the Anstis formula is used [32]. To determine the critical value of the stress concentration ratio, it is necessary to know the Young’s modulus and hardness of a given material.

Anstis formula:

(1) $$K_{\mathrm{IC}}=0.016\cdot (\;\frac{E}{HV}\;{)}^{0.5}\cdot \frac{P}{c^{1.5}}$$

where:

P—indenter load (N),

HV—Vickers hardness,

E—Young’s modulus of elasticity (MPa),

c = a + l—total crack length (m).

The tests of adhesion of coatings to the substrate and determination of other mechanical types of damage such as the indenter penetration depth, cracks, and the onset of delamination in the scratch path/scratch path profile were carried out by means of a scratch test using a Rockwell C-type diamond indenter with a radius of curvature of 100 µm at a constant load of 5, 10, 15, 20, and 25 N and progressive load of 0.03–30 N, using a multifunction measuring platform (Micro-Combi Tester, Switzerland) equipped with Anton Paar scratch test heads according to the standard [33]. The experiments were conducted on a multifunctional measuring platform (Micro-Combi Tester, Switzerland) equipped with Anton Paar scratch test heads, following the standard [33]. The samples, cross-sectioned and embedded in Durofast hard epoxy resin, were polished using standard metallographic procedures before testing. During the scratch test, the indenter traverses from the substrate through the coating into the resin. The specific parameters used for the scratch test are listed in Table 4. Two scratches were made on each sample to confirm the repeatability of the measurement results. The acoustic emission signal was recorded during the tests. In addition, microscopic analyses of the scratches were performed, which also made it possible to determine the strength of the coating and its resistance to destruction (cracking). The penetration depths of the indenter P_d_ were the parameters measured during the test. The damage to the coating/substrate system was detected and assessed through direct microscopic observation of the resulting scratch using light and scanning microscope. The normal force at which damage occurs is called the critical load. The critical load causing cohesive and adhesive cracks was determined; these cracks determine the quality of the coating–substrate bond.

Additionally, following the scratch test, the projected area of the cone-shaped fracture in the coating was determined using the formula Acn = L_x_·L_y_ for a constant load scratch force. This assessment provided insights into the coating’s cohesion and wear resistance, which were evaluated using a light microscope. The coefficient of friction was also measured in a scratch test on a multifunctional platform using a friction table and the same indenter as in the adhesion test with a progressive load applied on the penetrator (Rockwell C-type diamond indenter with a radius of curvature of 100 µm) with a linearly increasing pressure force applying load from 0 to 30 N on the penetrator. The scratch length was 1.4 mm and the indenter speed was 1.2 mm/min. During the measurement, the following parameters were recorded: applied force, friction force, friction coefficient, and scratch depth.

### 2.5. Corrosion Test

In order to determine the corrosion resistance of the tested coatings, the corrosion tests were performed in iron (III) chloride solution (in accordance with the standard ASTM A 923 [34], Test Method C) to simulate accelerated corrosion conditions of the coated material. For comparative purposes, the corrosion resistance of ductile iron samples (substrate) was determined. The samples for corrosion tests had the following dimensions: 15 × 15 mm^2^. The samples have been suitably protected so that corrosion only affects the coated surface. The corrosion solution was prepared by dissolving 55.1 g of iron chloride (FeCl_3_ × 6H_2_O) and 6.6 g of sodium nitrate (NaNO_3_) in 600 mL of distilled water, which corresponded to a concentration of about 5% FeCl_3_ and 1% NaNO_3_ by weight. The samples were immersed in a solution (250 mL per sample) at a constant temperature of 45 °C for 24 h. After the exposure, the samples were cleaned of corrosion products, dried, and weighed to an accuracy of ±0.1 mg using an electronic balance. The average corrosion rate (Vc) was determined using the formula:

(2) $$\mathrm{Vc}=\frac{∆m}{S.t}[g/m^{2}day]$$

where:

∆m—difference in sample mass before and after corrosion test (g),

S—sample area (m^2^),

t—duration of corrosion test (day).

After the calculations were performed, the samples were observed using light and scanning microscopy to assess the integrity of the coatings, the amount and intensity of corrosion changes, and the spread of subcoating corrosion. In order to analyze the phase composition of corrosion products, X-ray tests were performed using a diffractometer X’Pert Pro Panalytical (Malvern Panalytical Ltd., Cambridge, UK) in the angular range of 20–90° with CuK radiation.

## 3. Results and Discussion

### 3.1. Characterization of Coating Systems

**Figure 3:**
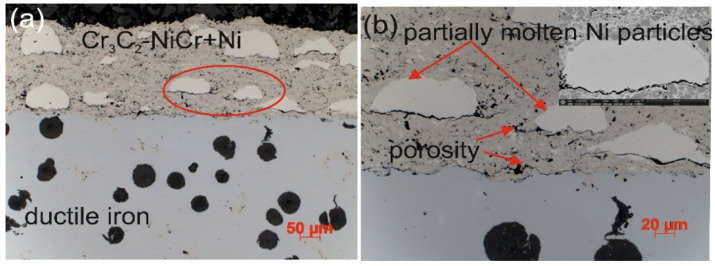
Cross-sectional microstructures of the (Cr_3_C_2_-NiCr+Ni)/ductile cast iron system at (**a**) low and (**b**) high magnifications.

The microstructure of the composite coating (Cr_3_C_2_-NiCr+Ni) sprayed onto a ductile cast iron substrate by the HVOF method (Figure 3) reflects the typical characteristics of the thermal spraying process. Carbide powder particles undergo intense plastic strain, forming layered, flattened grains. Fine chromium carbide particles of various sizes embedded in a nickel–chromium matrix were identified in the microstructure of the coating. Partially melted nickel particles were also observed, which became flattened and elongated parallel to the coating surface upon contact with the substrate. The coating demonstrates the ability to effectively fill substrate irregularities, which, in cross-section, reveals a solid mechanical connection resulting from the applied HVOF technology. The analyzed microstructure showed the presence of small amounts of oxides, pores, and micro-cracks. The oxides tend to accumulate locally within the coating or around partially melted nickel (Ni) particles. The characteristic properties of these oxides, such as high hardness, brittleness, and a lower coefficient of thermal expansion compared to the matrix, may contribute to the initiation and propagation of micro-cracks in the coating structure.

**Figure 4:**
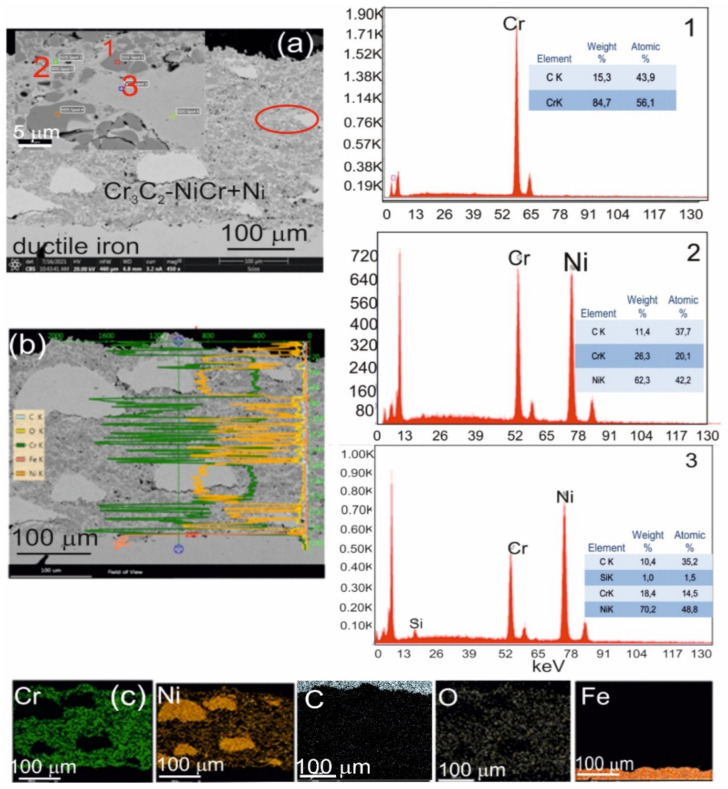
(**a**) Cross-sectional SEM micrographs of the (Cr_3_C_2_-NiCr+Ni)/ductile cast iron system with EDS spectra taken from the marked points 1, 2, and 3 (**b**) linear distribution of Cr, Ni, C, O, and Fe concentrations, and (**c**) elemental mapping of Cr, Ni, C, O, and Fe from the interface region.

Scanning electron microscopy (SEM) and energy-dispersive spectroscopy (EDS) analyses of the (Cr_3_C_2_-NiCr+Ni)/ductile iron coating system revealed a substantial refinement of chromium carbide grains—from approximately 40 μm in their initial state to 0.5–2.5 μm following the spraying process. This grain size reduction was observed both within the coating and at the interface between the coating and the substrate (Figure 4).

**Figure 5:**
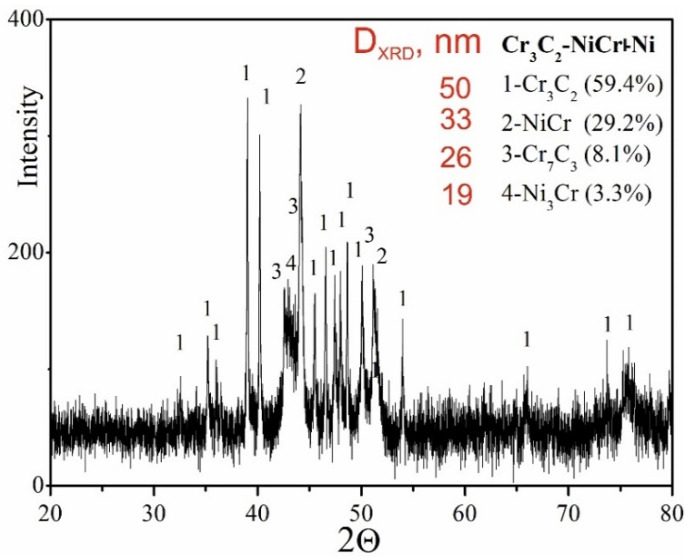
XRD pattern of the (Cr_3_C_2_-NiCr+Ni) coating.

The results of phase analysis of the composite coating (Cr_3_C_2_-NiCr+Ni), performed by X-ray diffraction (XRD), are shown in Figure 5. In addition to the Cr_3_C_2_ phase, the Cr_7_C_3_ phase was identified in the tested coating, formed as a result of partial decomposition of Cr_3_C_2_ under the action of a high-temperature spray jet acting on the powder grains, which is also characteristic of coatings without the participation of metallic particles [35]. The proportion of Cr_7_C_3_ phase was 8.3%, which indicates a low degree of decomposition of Cr_3_C_2_ carbide. Additionally, intermetallic phases such as NiCr (29.2%) and small amounts of Ni_3_Cr (3.3%) were detected in the composite coating. The diffraction lines of these phases showed no broadening, suggesting a low level of elastic–plastic deformation in their structure. While Cr_3_C_2_ possesses superior microhardness and nearly double the elastic modulus of Cr_7_C_3_, the formation of Cr_7_C_3_ within the NiCr matrix—arising from partial Cr_3_C_2_ decomposition—can beneficially affect the coating’s microstructure. This phase contributes to improved resistance against both wear and crack formation. Cr_7_C_3_ is known for its relatively high fracture toughness, with K_IC_ values reported between 2.64 and 4.53 MPa·m^1/2^ [36]. Additionally, the average crystallite size measurements of the phases indicate a nanocrystalline structure in the coating, which can play a key role in inhibiting crack development. The refinement of microstructural components enhances the bonding between carbide reinforcements and the metallic binder, leading to better ductility and mechanical integrity. The presence of carbides such as Cr_7_C_3_ and Cr_23_C_6_ further enhances the wear resistance of the coating, combining key properties resulting from both the fine-grained structure and the specific carbide characteristics. This combination of features makes the coating more resistant to mechanical loads and wear processes in demanding operating conditions.

**Figure 6:**
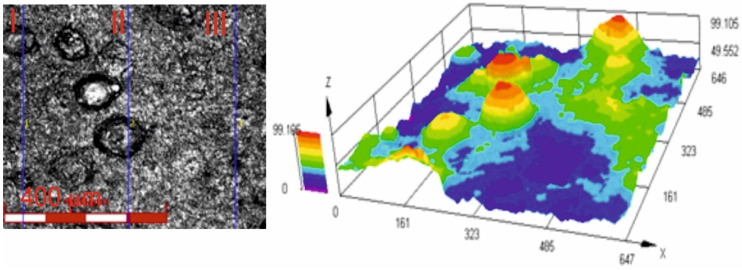
Three-dimensional view of the surface of the (Cr_3_C_2_-NiCr+Ni) coating recorded using a confocal laser scanning microscope.

Figure 6 shows an image of the surface roughness measurement site, obtained using a confocal laser microscope for the Cr_3_C_2_-NiCr+Ni coating at 200× magnification, along with a three-dimensional image of the surface of this coating. The image analysis enabled a precise examination of the geometric structure of the coating surface. The results of roughness parameter measurements, such as R_a_ and R_z_, for the composite coating (Cr_3_C_2_-NiCr+Ni) are summarized in Table 5. For comparison, the average roughness values R_a_ and R_z_ for the standard Cr_3_C_2_-NiCr coating were 4.8 ± 1.1 μm and 26.9 ± 4.9 μm, respectively [35]. The greater surface roughness of the composite coating is primarily due to its composite structure. This is likely a result of the crystallization process of hemispherical nickel (Ni) particles and their island-like arrangement in the coating matrix. The presence of distinct sharp peaks on these particles further contributes to the roughness. The larger the dimensions of the Ni particles, the more they affect the geometric properties of the coating surface. These factors are important when interpreting scratch test results, particularly in the context of their influence on adhesion and coating wear rate. It is also worth noting that such a surface topography of the coating may contribute to improving its abrasion resistance. The average porosity of Cr_3_C_2_-NiCr [35] and (Cr_3_C_2_-NiCr+Ni) coatings sprayed onto ductile cast iron, determined from tomographic measurements, was 3.6 ± 0.8% and 2.4 ± 0.6%, respectively. The low porosity observed in composite coatings is primarily a result of the significant surface area covered by partially melted nickel (Ni) particles, which contribute to the formation of a tightly packed material structure.

The results of microhardness and Young’s modulus tests for the coating system (Cr_3_C_2_-NiCr+Ni)/ductile cast iron are presented in Table 6. The coatings containing nickel (Ni) particles were characterized by lower hardness and Young’s modulus values compared to coatings without metallic particles [35]. This is due to the presence of soft Ni particles in the coating microstructure, which results in a reduction of both hardness and stiffness. The average H_IT_ and E_IT_ values for the standard Cr_3_C_2_-NiCr coating system were 10.9 ± 1.97 GPa and 203.77 ± 11.91 GPa, respectively, while for the composite coating system (Cr_3_C_2_-NiCr+Ni), they were 7.70 ± 2.42 GPa and 191.69 ± 18.58 GPa, respectively. The highest hardness values were observed in the central part of the cross-section of the coatings, at a distance of approximately 100 µm from the substrate. Maximum values of Young’s modulus were also recorded in this area, which can be attributed to the strain hardening effect occurring during the spraying process, better coating cohesion, and significant refinement of carbide grains. The lowest microhardness and Young’s modulus values were found in regions containing nickel (Ni) particles, as these particles are softer than the surrounding matrix. Overall, the microhardness of the Cr_3_C_2_-NiCr and (Cr_3_C_2_-NiCr+Ni) coatings generally increases as the distance from the substrate/coating interface grows. This variation in hardness is linked to changes in the chemical composition and phase structure of the carbide coating. The decrease in hardness near the coating/substrate interface, along with a reduced bond between the metallic particles and the matrix, may be attributed to decarburization and oxidation of certain alloying elements during the thermal spraying process and the subsequent cooling from molten to room temperature.

The fracture toughness results for Cr_3_C_2_-NiCr and (Cr_3_C_2_-NiCr+Ni) coatings are presented in Table 7 and Table 8 together with images of indentations made during indentation microhardness measurements, which were used to determine the crack lengths and indentation diagonals. Based on the obtained values of instrumental microhardness (H_IT_) and the modulus of elasticity (E_IT_), the critical fracture toughness coefficient (K_IC_) was calculated. The results presented in Table 7 and Table 8 confirm that Young’s modulus significantly influences the coating’s fracture toughness. However, when assessing this property, the hardness and porosity of the coating are equally important. The fracture toughness coefficient of the coating without Ni particles (Cr_3_C_2_-NiCr) at a load of 10–20 N was in the range of 2.4–0.8 M Nm^3/2^, whereas for the (Cr_3_C_2_-NiCr+Ni) composite coating at a load of 5–15 N, it ranged from 3.2 to 1.9 MNm^3/2^ in the matrix and from 1.8 to 1.2 MNm^3/2^ in the areas containing Ni particles. Higher values of K_IC_ for composite coatings at lower loads (5–15 N), resulting from shorter cracks and a higher modulus of elasticity to hardness ratio (E/H), indicate an increased capacity for plastic strain compared to coatings without metallic particles. The presence of metallic particles (Ni) helps improve the plasticity of the coating, which limits crack propagation. In addition, higher K_IC_ values may result from the lower porosity of the composite coatings. The measurements of the fracture toughness coefficient for composite coatings showed greater variability of the results, which is a consequence of their inhomogeneous microstructure. It is also important that the process of decarburization and dissolution of Cr_3_C_2_ during spraying leads to the the formation of a submicron-sized phase, which affects the mechanical properties of the matrix and thus increases its crack resistance [37]. The analysis of the results of microhardness (H_IT_), Young’s modulus (E_IT_), and fracture toughness coefficient (K_IC_) measurements in cross-sections of coating systems shows that the introduction of metallic particles locally reduces the hardness of the coating, which results in a reduction of its susceptibility to brittle fracture. The metal particles, constituting a soft phase compared to the brittle chromium carbide grains, promote plastic strain of the coating. This makes it possible to obtain coatings that combine high hardness with improved plasticity, which consequently increases their resistance to wear and cracking. Reducing the microhardness of the coating is also associated with a reduction in the modulus of elasticity, which further improves the coating’s ability to undergo plastic strain. It is worth emphasizing that the fracture toughness of the coatings depends not only on their mechanical properties, but also on the morphological parameters of the microstructure. The key factors include the content of Ni particles, their size and shape, the strength of the adhesive bond between Ni particles and the NiCr matrix, as well as the magnitude of internal stresses that can lead to decohesion of these particles [38].

**Table 7:** Indentation fracture toughness measurements of the Cr_3_C_2_-NiCr coating under loads of 5, 10, 15, and 20 N.

Indenter Print Image	Load (N)	H_IT_ (GPa)	E_IT_ (GPa)	K_IC_ (MN m^−3/2^)	Average K_IC_ (MN m^−3/2^)
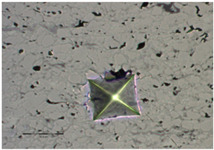	5	9.30	190.11		No cracks
		10.12	194.15		
		10.36	200.94		
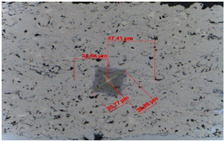	10	10.45	184.16	2.06	2.37 ± 0.06
		9.86	180.94	2.59	
		8.23	177.20	2.45	
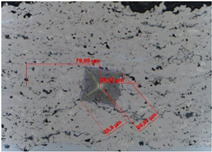	15	9.86	166.68	0.92	1.29 ± 0.09
		9.07	166.26	1.45	
		9.54	168.16	1.52	
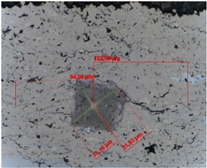	20	9.22	168.04	0.88	0.81 ± 0.01
		8.56	165.33	0.81	
		9.19	159.42	0.73	

**Figure 7:**
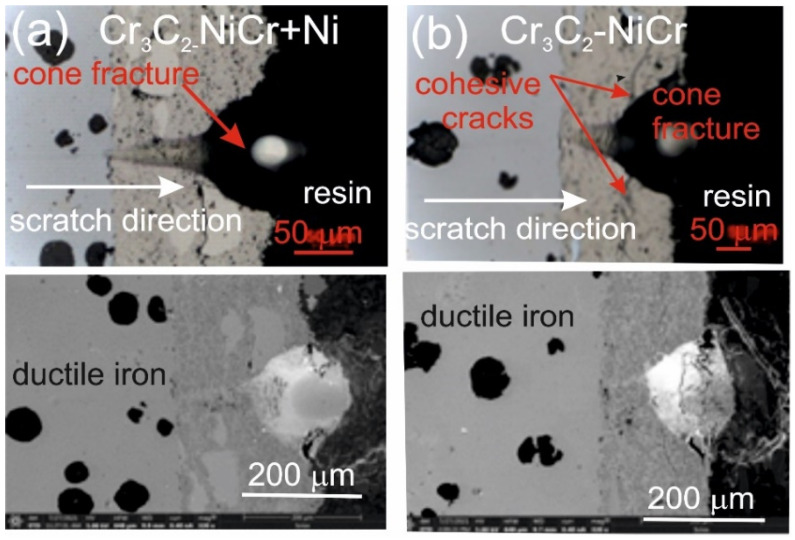
LM/SEM micrographs showing cone-shaped fractures occurring during the scratch bond strength test under progressive loading (0.03–3 N) for the following coating systems: (**a**) (Cr_3_C_2_-NiCr+Ni)/ductile cast iron and (**b**) Cr_3_C_2_-NiCr/ductile cast iron.

**Figure 8:**
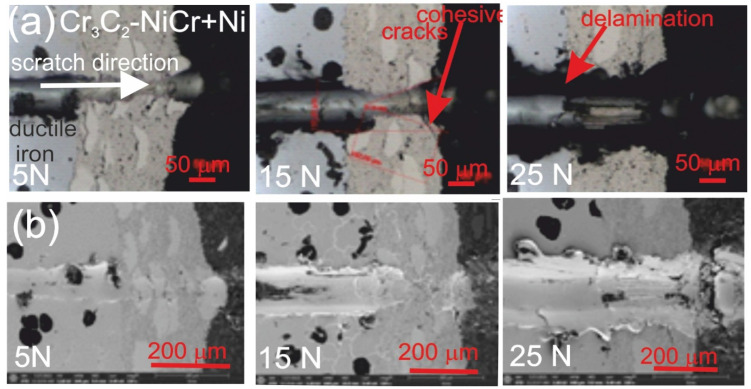
LM/SEM micrographs showing cone-shaped fractures occurring during the scratch bond strength test under constant loads of 5, 15, and 25 N for the (Cr_3_C_2_-NiCr+Ni)/ductile cast iron coating system. (**a**) LM micrographs; (**b**) SEM micrographs.

Figure 7 and Figure 8 show the scratch trace in the (Cr_3_C_2_-NiCr+Ni)/ductile iron and Cr_3_C_2_-NiCr/ductile cast iron coating systems under the application of constant and progressive loads. The analysis of the results showed that the scratch penetration depth depends on the type of load applied and the coating microstructure. During the scratch test, parallel measurements of the scratch penetration depth were performed, which allowed for the assessment of the resistance of the tested coating systems to scratching and plastic strain. The results of penetration depth measurements for various coating systems are summarized in Table 9. These data enable a comparison of the behavior of standard Cr_3_C_2_-NiCr and composite (Cr_3_C_2_-NiCr+Ni) coatings, indicating a significant influence of the presence of metallic particles on the scratch characteristics. In the case of coatings with the addition of metallic particles, more favourable values of the indenter penetration depth were observed during the scratch test, which indicates their higher scratch resistance in comparison to coatings without these particles. Analysis of the results showed that with the increase in load, the indenter penetration depth gradually increases in all tested coating systems. The coatings enriched with metallic particles showed better tribological properties, as confirmed by the smaller indenter penetration depth in the load range tested. The presence of hard carbide phases and other nickel-based phases effectively limits the propagation of micro-damages and micro-cuts, minimising tears along the resulting cracks.

**Table 8:** Indentation fracture toughness measurements of the (Cr_3_C_2_-NiCr+Ni) coating under loads of 5, 10, and 15 N.

Indenter Print Image	Load (N)	H_IT_ (GPa)	E_IT_ (GPa)	K_IC_ (MN m^−3/2^)	Comments
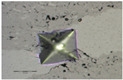	5	8.76	187.86	3.24	Matrix
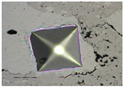		3.42	143.44		No cracks
** 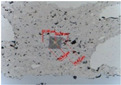 **		5.13	165.58		No cracks
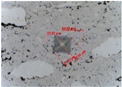	10	9.75	178.15	2.71	Matrix
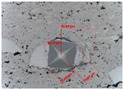		5.04	166.77	1.84	Ni-particle
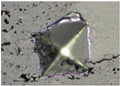		6.53	163.84		No cracks
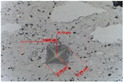	15	9.67	165.56	1.89	Matrix
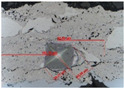		6.43	154.99	1.15	Ni-particle
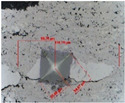		4.82	145.01	1.05	Interface

Such structural properties contribute to improved abrasion resistance, which makes coatings with the addition of metallic particles more resistant to intense mechanical loads and wear processes occurring in operating conditions requiring high mechanical strength [37].

### 3.2. Wear and Friction Behavior

The assessment of the adhesion of the coating to the substrate was performed based on the analysis of microscopic scratch images obtained during tests carried out at progressive loads in the range of 0.03–30 N and at constant loads of 5, 10, 15, 20, and 25 N. The obtained scratch images of the tested coating systems are presented in Figure 7 and Figure 8. The scratch trace on the cross-section of the (Cr_3_C_2_-NiCr+Ni)/ductile cast iron coating system indicates that the cracks propagate mainly in the areas containing nickel (Ni) particles. Due to their plasticity, Ni particles take over the propagation of cracks, extinguishing the energy of their spreading.

This process occurs by inhibiting the movement of the crack and deflecting its direction, which helps to increase the mechanical resistance of the coating. During the scratch test with progressively increasing load, different damage mechanisms were observed in the cross-sections of the coating systems. The cohesive cracks dominated in the coatings devoid of metallic particles and propagated parallel to the substrate-coating interface, inside the coating itself. For the coatings containing metallic particles, the cracks occurred primarily at the metallic particle/matrix interface but did not propagate further into the coating microstructure. The presence of Ni particles plays a key role in limiting crack growth, which significantly improves the mechanical integrity of the coating. These particles act as plastic buffers that capture and reduce local stresses in places where cracks could develop. In this way, they increase the resistance of the coating to mechanical damage, such as scratches and delamination. In the case of the (Cr_3_C_2_-NiCr+Ni)/ductile cast iron coating system, the calculated Acn (projected area of the cone) values increased with the increasing load in the range of 10–20 N (Table 10). This result confirms the good scratch bond strength of this system in the tested load range. The characteristic cone-shaped fractures occurred within the coating and suggested a cohesive damage mechanism in the coating/substrate system at loads in the range of 10–20 N. This means that the damage initiated in this load range developed mainly within the coating itself, indicating its high structural integrity and resistance to detachment from the substrate. Larger cracks were observed around the scratch in the coating system containing metallic particles (Ni) at a maximum load of 25 N. This phenomenon led to delamination of the coating from the substrate, which indicates an adhesive damage mechanism under these loading conditions.

The addition of Ni particles significantly increases the mechanical strength of the coatings by improving their scratch resistance and delaying the initiation of adhesive damage mechanisms. In composite coatings, the delamination moment shifts toward higher loads, which indicates increased structural strength compared to coatings without the Ni particles added [35]. Additionally, based on microscopic images obtained after the scratch tests, a detailed analysis of the proportions of different forms of damage in the tested coating system was performed. The identified damage types included no cracks, cohesive cracks, and adhesive cracks as illustrated in Annex A to ISO 27307:2015(E) [33]. The adhesive cracks occurred at the coating interface: for the Cr_3_C_2_-NiCr/ductile cast iron system at a load of 20 N and for the (Cr_3_C_2_-NiCr+Ni)/ductile cast iron system at 25 N. The cohesive cracks occurred inside the coating: for Cr_3_C_2_-NiCr/ductile cast iron above 5 N and for (Cr_3_C_2_-NiCr+Ni)/ductile cast iron above 10 N (Table 11). The results show that the type of coating and load affect the type of damage, which emphasizes the need to optimize the composition and microstructure of coatings in order to increase their durability and mechanical resistance.

**Figure 9:**
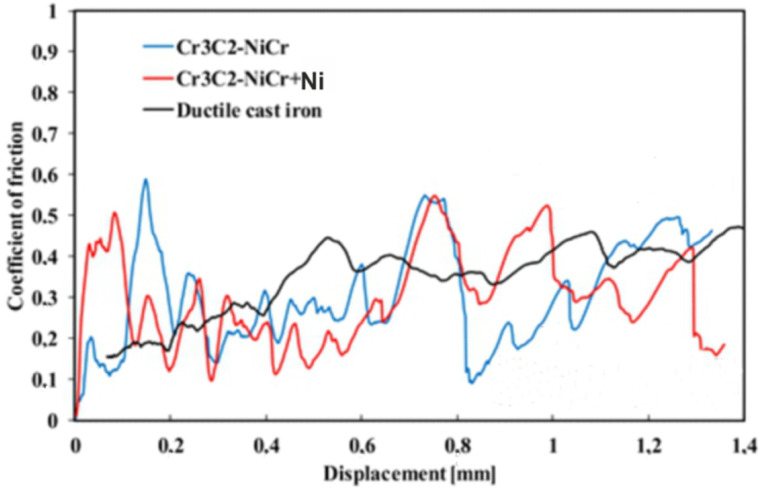
Variation of the friction coefficient with sliding distance for the following coatings: (Cr_3_C_2_-NiCr+Ni), Cr_3_C_2_-NiCr, and ductile cast iron, along with optical micrographs of the wear track after the scratch test.

The evolution of the friction coefficient during the scratch test of the coating surface at a progressive load (0.03–30 N) over a length of 1.4 mm is shown in Figure 9. The coatings demonstrated similar tribological characteristics, but clear fluctuations in the friction coefficient were observed depending on the phase composition. For the composite coating (Cr_3_C_2_-NiCr+Ni), the fluctuations ranged from 0.09 to 0.55, with an average value of 0.31, while for the Cr_3_C_2_-NiCr coating, the fluctuations were in the range of 0.11–0.59, with an average friction coefficient of 0.30. The reduction in the amplitude of friction coefficient fluctuations for the composite coating was due to the presence of Ni particles, which, through more frequent contact with the indenter, increased the friction resistance, which resulted in a slight increase in the average friction coefficient to 0.31. The Ni particles, being a metallic material, contributed to increased friction and intensified wear of the coating, which was associated with increased roughness of its surface. The higher surface roughness of the composite coating could also contribute to its higher wear resistance, because the indenter deformed or crushed the micro-irregularities on the surface during the test, leading to friction force fluctuations. In contrast, the Cr_3_C_2_-NiCr coating showed a lower friction coefficient, which was probably due to its higher hardness and lower surface roughness. The friction coefficient values for the coatings tested were significantly lower than for the ductile iron substrate (µ = 0.40), which confirms the improvement of the tribological properties of the coated cast iron.

### 3.3. Corrosion Behavior

**Figure 10:**
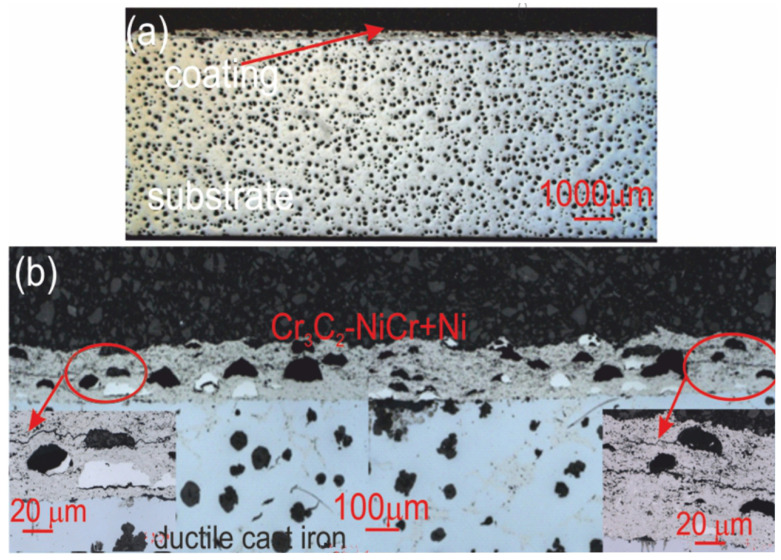
Cross-sectional LM micrographs of the corroded (Cr_3_C_2_-NiCr+Ni)/ductile cast iron specimen after corrosion testing in an iron chloride solution. (**a**) low magnification, (**b**) high magnification.

**Figure 11:**
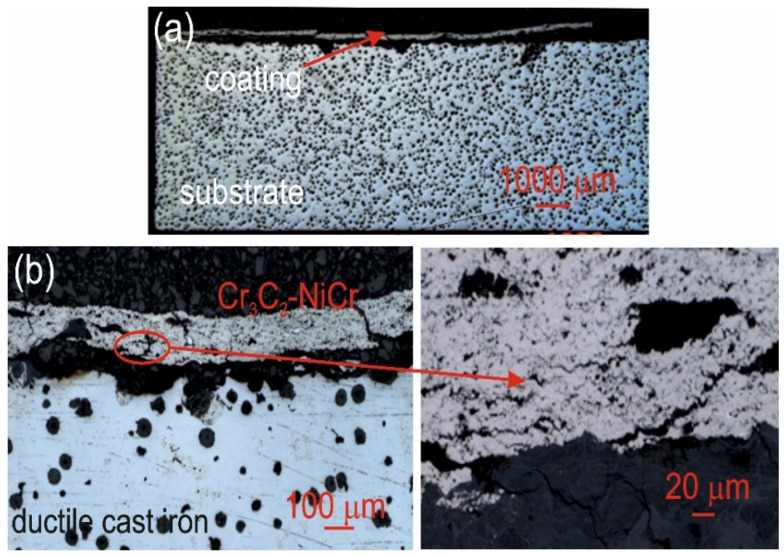
Cross-sectional LM micrographs of the corroded Cr_3_C_2_-NiCr/ductile cast iron specimen after corrosion testing in an iron chloride solution. (**a**) low magnification, (**b**) high magnification.

Table 12 summarizes the average corrosion rates and key results from the corrosion resistance tests on the materials: ductile cast iron, Cr_3_C_2_-NiCr/ductile cast iron, and (Cr_3_C_2_-NiCr+Ni)/ductile cast iron. The primary cause of corrosion in the coatings was the selective dissolution of the metal matrix (NiCr), which is more anodic than the chromium carbide particles. Examination of the cross-sectional micrographs of the coating systems—(Cr_3_C_2_-NiCr+Ni)/ductile cast iron and Cr_3_C_2_-NiCr/ductile cast iron (Figure 10 and Figure 11)—after corrosion testing showed significant differences in their resistance.

For the composite coatings (Cr_3_C_2_-NiCr+Ni), the corrosion process was selective, concentrating on nickel particles. The formation of dark deposits on the Ni particles and cracks parallel to the coating/substrate interface were observed. For the coatings without the addition of metallic particles (Cr_3_C_2_-NiCr), local clusters of pits, numerous cracks perpendicular to the interface, and complete delamination of the coating from the substrate occurred. The increased porosity of the Cr_3_C_2_-NiCr coating accelerated the corrosion process by creating electrochemical potential differences between the corrosion pores and the matrix, which led to intensified material degradation. Microscopic examination of the surface of the composite coating (Cr_3_C_2_-NiCr+Ni) after exposure to a corrosive environment revealed the presence of volumetric corrosion areas and micro-cracks that facilitated the penetration of the corrosive solution. As a result, the pits were gradually filled with corrosion products, which led to further degradation of the coating structure.

**Figure 12:**
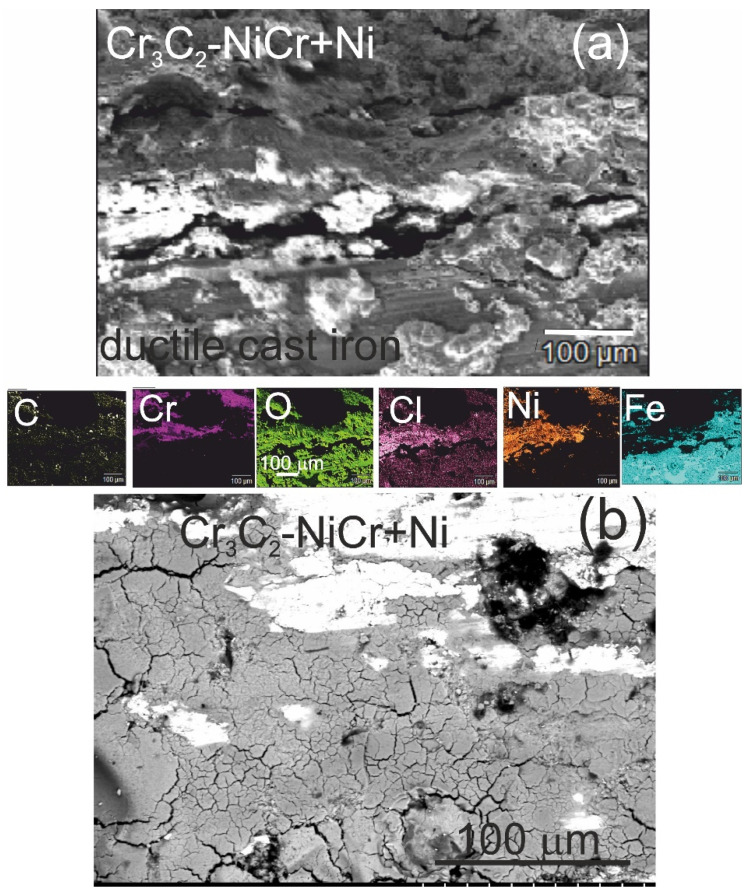
SEM micrographs and corresponding EDS elemental mapping of a corroded (Cr_3_C_2_-NiCr+Ni)/ductile cast iron specimen after corrosion testing in an iron chloride solution: (**a**) cross-sectional image and (**b**) surface image.

The analysis of the chemical composition of corrosion products on the cross-section (Figure 12a) and surface (Figure 12b) of the (Cr_3_C_2_-NiCr+Ni)/ductile cast iron coating system using EDS revealed the presence of carbon, oxygen, chromium, nickel, iron, and chlorine, suggesting the formation of complex layers of corrosion products.

**Figure 13:**
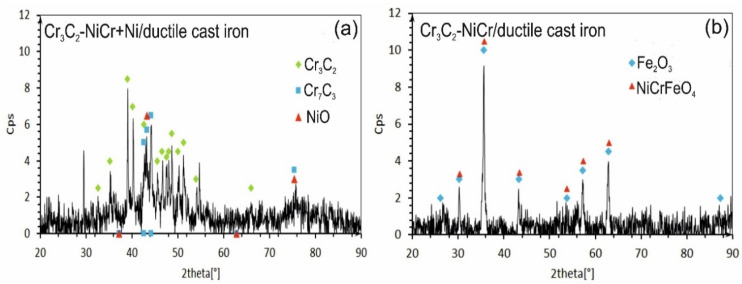
XRD analysis of corroded specimens after corrosion testing in an iron chloride solution: (**a**) (Cr_3_C_2_-NiCr+Ni)/ductile cast iron and (**b**) Cr_3_C_2_-NiCr/ductile cast iron.

The X-ray diffraction (XRD) tests of the phase composition of the composite coating revealed the presence of Cr_3_C_2_ and Cr_7_C_3_ and a small amount of nickel oxide (NiO). After exposure to a corrosive environment, the intensity of the peaks associated with the carbide phase decreased, indicating partial degradation of this phase. Moreover, the lack of peaks characteristic of the NiCr phase suggests its complete transformation into NiO (Figure 13a). The (Cr_3_C_2_-NiCr+Ni) composite coating showed superior corrosion resistance, which was attributed to its ability to form protective layers of corrosion products, such as NiO, that filled the pores and limited further degradation of the material. In turn, for the Cr_3_C_2_-NiCr coating (Figure 13b), the dominant chemical compounds on the surface were iron oxide (Fe_3_O_4_) and Ni-Cr-Fe oxide (NiCrFeO_4_), which formed as a result of the coating delamination process. The interfacial corrosion at the coating–substrate interface, supported by electrochemical potential differences, promoted the formation of galvanic micro-cells, which led to intensified degradation. The analysis of the corrosion test carried out in iron chloride solution shows that the corrosion mechanisms in the coating systems tested are complex. Porosity, interphase boundaries, carbide distribution in the metal matrix, and oxide inclusions play a key role in the corrosion process. The (Cr_3_C_2_-NiCr+Ni) composite coatings demonstrate higher corrosion resistance due to their ability to form protective layers of corrosion products, which results in limiting further degradation of the material.

## 4. Conclusions

From the research conducted and the analysis results, the following conclusions can be drawn:

1. The composite coating (Cr_3_C_2_-NiCr+Ni), applied using the HVOF technique, exhibits a dense, layered microstructure with no visible defects at the interface between the coating and substrate. The microstructure contains elongated, plastically deformed Ni-Cr alloy particles, fine Cr_3_C_2_ particles (ranging from 0.5 to 2.5 µm), and partially molten nickel particles with a flattened shape. Phase analysis identified the presence of Cr_7_C_3_ and Ni_3_Cr phases, which contribute to the reinforcement of the nickel–chromium matrix. The relatively low decomposition of Cr_3_C_2_ into Cr_7_C_3_ (8.3%) indicates the carbide’s high chemical stability during the spraying process.
2. The modification of the coating composition by adding nickel particles improves its mechanical, tribological, and corrosion properties. The reduction of microhardness (H_IT_) and the modulus of elasticity (E_IT_) increases the plasticity of the coating, limiting crack propagation and delaying the delamination process, which improves its structural integrity. The presence of nickel particles strengthens the coating due to synergistic interaction with Cr_3_C_2_, resulting in increased resistance to mechanical wear and scratches. The coating also demonstrates improved corrosion resistance due to the formation of protective oxide layers (NiO).
3. (Cr_3_C_2_-NiCr+Ni) coatings deposited on cast iron substrates by the HVOF method are an effective solution for applications requiring high mechanical strength, resistance to cracking, wear and corrosion. The synergistic effect between hard chromium carbide particles and ductile nickel particles ensures high operating efficiency, especially in environments exposed to intense mechanical and corrosive impacts.

## Figures and Tables

**Table 1:** Chemical composition of EN-GSJ-500-7.

Chemical Composition, wt.%									
C	Si	Mn	P	S	Cr	Ni	Mg	Cu	Fe
3.61	2.29	0.45	0.045	0.009	0.03	0.01	0.057	0.75	rest

**Table 2:** Mechanical properties of EN-GSJ-500-7.

Tensile Strength (MPa)	Conventional Yield Point (MPa)	Elongation (%)	Hardness (HB)	Elastic Modulus (GPa)
500	340	7	230	169

**Table 3:** HVOF spraying parameters as sprayed Cr_3_C_2_-NiCr coatings.

Gun Movement Speed (mm/s)	Oxygen (L/min)	Kerosene (L/h)	Powder Feed Rate (g/min)	Powder Feed Gas (L/min)	Spraying Distance (mm)
583	850	24	65	Nitrogen, 9.5	370

**Table 4:** Scratch test parameters.

Intender	Scratch Mode	Load (N)	Scratch Length (mm)	Scratch Speed (mm/s)
Rockwell C, 100 μm	Continuous load	5, 10, 15, 20, 25	1.4	1.2
	Progressive load	0.03–30	0.35	1.2

**Table 5:** Surface roughness parameters Ra, Rz for (Cr_3_C_2_-NiCr+Ni) composite coating.

Coating	Measurement	Ra (µm)	Rz (µm)
(Cr_3_C_2_-NiCr+Ni)	1	9.7	41.6
	2	14.7	69.8
	3	7.9	37.3
	Average value (µm)	10.8 ± 3.5	49.6 ± 17.6

**Table 6:** Indentation hardness (H_IT_) and Young’s modulus (E_IT_) values of the (Cr_3_C_2_-NiCr+Ni)/ductile cast iron coating system.

Indenter Print Image	Region	H_IT_ (GPa)	E_IT_ (GPa)	Average H_IT_ (GPa)	Average E_IT_ (GPa)
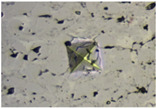	Matrix (upper area)	7.88	205.68	8.54 ± 0.80	193.44 ± 10.93
		9.68	194.32		
		8.06	180.32		
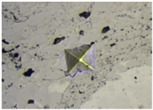	Matrix (center)	11.54	205.68	10.11 ± 0.95	198.59 ± 13.03
		9.24	209.79		
		9.56	180.32		
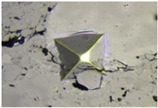	Metallic particle	3.84	181.74	3.56 ± 0.08	151.56 ± 21.75
		3.62	141.63		
		3.21	131.30		
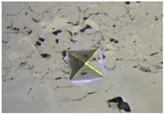	Interface	8.45	208.92	7.04 ± 1.39	198.15 ± 8.88
		6.73	187.16		
		5.83	198.36		

**Table 9:** Penetration depth measurements in the scratch path under constant and progressive loading for Cr_3_C_2_-NiCr/ductile cast iron [35] and (Cr_3_C_2_-NiCr+Ni)/ductile cast iron systems.

Coating System	Load (N)					
	5	10	15	20	25	0.03–30
	Depth Penetration Pd (μm)					
Cr_3_C_2_-NiCr/ductile cast iron	8	15	22	34	48	37 at approx. 25 N
(Cr_3_C_2_-NiCr+Ni)/ductile cast iron	7	14	21	30	46	41 at approx. 25 N

**Table 10:** Averaged scratch bond test results of the (Cr_3_C_2_-NiCr+Ni)/ductile cast iron coating system.

Coating System	Load (N)	Lx (µm)	Ly (µm)	A_cn_ × 10^−3^ (mm^2^)
(Cr_3_C_2_-NiCr+Ni)/ductile cast iron	5	-	-	-
	10	104.12	110.46	11.51
	15	182.58	117.38	21.43
	20	206.29	217.28	44.82
	25	delamination		

**Table 11:** Percentage distribution of failure types under constant load (no cracks, cohesive cracks, adhesive cracks) based on ISO 27307:2015 Standard: Thermal spraying—Evaluation of adhesion/cohesion of thermal sprayed ceramic coatings by transverse scratch testing [33].

Coating System	Load (N)	No Crack (%)	Cohesive Crack (%)	Adhesive Crack (%)	Maximum Load at Which Cohesive Cracks Appears	Maximum Load at Which Adhesive Cracks Appears
Cr_3_C_2_-NiCr/ductile cast iron	5	85	15	0	over 5 N	
	10	70	30	0		
	15	50	50	0		
	20	0	0	100		delamination
(Cr_3_C_2_-NiCr+Ni)/ductile cast iron	5	100	0	0		
	10	90	10	0	over 10 N	
	15	80	20	0		
	20	50	50	0		
	25	0	0	100		delamination

**Table 12:** Basic results from the corrosion resistance test of the tested materials: ductile cast, Cr_3_C_2_-NiCr/ductile cast iron, and (Cr_3_C_2_-NiCr+Ni)/ductile cast iron.

Materials	Surface Area (mm^2^)	Weight Before Test (g)	Weight After Test (g)	Mass Loss (g)	Corrosion Rate (g/m^2^doba)
Ductile cast iron	102	25.44750	25.42659	0.02091	205.65
Cr_3_C_2_-NiCr/ductile iron	109	25.48380	25.47234	0.01146	105.14
(Cr_3_C_2_-NiCr+Ni)/ductile iron	113	27.21759	27.21132	0.00627	55.49

## Data Availability

The original contributions presented in this study are included in the article. Further inquiries can be directed to the corresponding author.

## References

[1] Davis J.R. (2004). Handbook of Thermal Spray Technology.

[2] Pawlowski L. (2008). The Science and Engineering of Thermal Spray Coatings.

[3] Straffelini G., Federici M. (2020). HVOF cermet coatings to improve sliding wear resistance in engineering systems. Coatings.

[4] Xie M., Zhang S., Li M. (2013). Comparative investigation on HVOF sprayed carbide-based coatings. Appl. Surf. Sci..

[5] Reddy N.C., Kumar B.S.A., Reddappa H.N., Ramesh M.R., Koppad P.G., Kord S. (2018). HVOF sprayed Ni_3_Ti and Ni_3_Ti+ (Cr_3_C_2_+20NiCr) coatings: Microstructure, microhardness and oxidation behavior. J. Alloys Compd..

[6] Kunioshi C.T., Correa O.V., Ramanathan L.V. (2006). High temperature oxidation and erosion-oxidation behaviour of HVOF sprayed Ni-20Cr, WC-20Cr-7Ni and Cr_3_C_2_-Ni-20Cr coatings. Surf. Eng..

[7] Matikainen V., Bolelli G., Koivuluoto H., Sassatelli P., Lusvarghi L., Vuoristo P. (2017). Sliding wear behaviour of HVOF and HVAF sprayed Cr_3_C_2_-based coatings. Wear.

[8] Bolelli G., Berger L.M., Borner T., Koivuluoto H., Matikainen V., Lusvarghi L., Lyphout C., Markocsan N., Nylen P., Sassatelli R. (2016). Sliding and abrasive wear behaviour of HVOF- and HVAF-sprayed Cr_3_C_2_-NiCr hardmetal coatings. Wear.

[9] Formanek B., Szymański K., Szczucka-Lasota B. (2005). New generation of protective coatings intended for the power industry. J. Mater. Process. Technol..

[10] Berger L.M. (2015). Application of hardmetals as thermal spray coatings. Int. J. Refract. Met. Hard Mater..

[11] Jin D., Yang F., Zou Z., Gu L., Zhao X., Guo F., Xiao P. (2016). A study of the zirconium alloy protection by Cr_3_C_2_–NiCr coating for nuclear reactor application. Surf. Coat. Technol..

[12] Venkatesh L., Pitchuka S.B., Sivakumar G., Gundakaram R.C., Joshi S.V., Samajdar I. (2017). Microstructural response of various chromium carbide based coatings to erosion and nano impact testing. Wear.

[13] Matthews S., Berger L.M. (2016). Long-term compositional/microstructural development of Cr_3_C_2_-NiCr coatings at 500 °C, 700 °C and 900 °C. Int. J. Refract. Met. Hard Mater..

[14] Singh H., Kaur M., Prakash S. (2016). High-temperature exposure studies of HVOF-Sprayed Cr_3_C_2_-25 (NiCr)/(WC-Co) Coating. J. Therm. Spray Technol..

[15] Varis T., Suhonen C., Calonius O., Cuban J., Pietola M. (2016). Optimization of HVOF Cr_3_C_2_–NiCr fatigue performance NiCr coating for increase. Surf. Coat.Technol..

[16] Prudenziati M., Gazzadi G.C., Medici M., Dalbagni G., Caliari M. (2010). Cr_3_C_2_-NiCr HVOF-sprayed coatings: Microstructure and properties versus powder characteristics and process parameters. J. Therm. Spray Technol..

[17] Matthews S. (2015). Carbide dissolution/carbon loss as a function of spray distance in unshrouded/shrouded plasma sprayed Cr_3_C_2_-NiCr coatings. J. Therm. Spray Technol..

[18] Li C., Ji G., Wang Y., Sonoya K. (2002). Dominant effect of carbide rebounding on the carbon loss during high velocity oxy-fuel spraying of Cr_3_C_2_-NiCr. Thin Solid Films.

[19] Yuan J., Ma C., Yang S., Yu Z., Li H. (2016). Improving the wear resistance of HVOF sprayed WC-Co coatings by adding submicron-sizedWC particles at the splats’ interfaces. Surf. Coat. Technol..

[20] Roy M., Pauschitz A., Bernardi J., Koch T., Franek F. (2006). Microstructure and mechanical properties of HVOF sprayed nanocrystalline Cr_3_C_2_-25 (Ni20Cr) coating. J. Therm. Spray Technol..

[21] Chen J., Zhao X., Zhou H., Chen J., An Y., Yan F. (2014). Microstructure and tribological property of HVOF sprayed adaptive NiMoAl–Cr_3_C_2_–Ag composite coating from 20 °C to 800 °C. Surf. Coat. Technol..

[22] Zhang Y., Chong K., Liu Q., Bai Y., Zhang Z., Wu D., Zou Y. (2021). High-temperature tribological behavior of thermally-treated supersonic plasma sprayed Cr_3_C_2_-NiCr coatings. Int. J. Refract. Met. Hard Mater..

[23] Picas J.A., Punset M., Manergues E., Martin E., Baile M.T. (2015). Microstructural and tribological studies of as-sprayed and heat-treated HVOF Cr_3_C_2_–CoNiCrAlY coatings with a CoNiCrAlY bond coat. Surf. Coat. Technol..

[24] He B., Zhang L., Yun X., Wang J., Zhou G., Chen Z., Yuan X. (2022). Comparative study of HVOF Cr_3_C_2_–NiCr coating with different bonding layer on the interactive behavior of fatigue and corrosion. Coatings.

[25] Janka L., Norpoth J., Trache R., Berge L.M. (2016). Influence of heat treatment on the abrasive wear resistance of a Cr_3_C_2_–NiCr coating deposited by an ethene-fuelled HVOF spray process. Surf. Coat. Technol..

[26] Gariboldi E., Rovatti L., Lecis N., Mondora L., Mondora G.A. (2016). Tribological and mechanical behaviour of Cr_3_C_2_–NiCr thermally sprayed coatings after prolonged aging. Surf. Coat. Technol..

[27] Mazaheri Y., Malmir R., Jalilvand M.M., Sheikhi M., Heidarpour A. (2022). Mechanical properties and tribological performance of A356/Cr_3_C_2_-NiCr surface composite developed by high-velocity oxy-fuel and post friction stir processing treatment. Surf. Interfaces.

[28] Wang K., Hong S., Wei Z., Hu N., Cheng J., Wu Y. (2021). Long-term corrosion behavior of HVOF sprayed Cr_3_C_2_-NiCr coatings in sulfide-containing 3.5 wt.% NaCl solution. J. Mater. Res. Technol..

[29] Zavareh M.A., Sarhan A.A.D., Bushroa A.R., Basirun W.J. (2015). The tribological and electrochemical behavior of HVOF-sprayed Cr_3_C_2_-NiCr ceramic coating on carbon steel. Ceram. Int..

[30] Magnani M., Suegama P.H., Espallargas N., Fugivara C.S., Dosta S., Guilemany J.M., Benedetti A.V. (2009). Corrosion and wear studies of Cr_3_C_2_-NiCr-HVOF coatings sprayed on AA7050 T7 under cooling. J. Therm. Spray Technol..

[31] Hassanzadeh-Tabrizi S.A. (2023). Precise calculation of crystallite size of nanomaterials: A review. J. Alloys Compd..

[32] Anstis G., Chantikul P., Lawn B., Marshall D. (1981). A Critical Evaluation of Indentation Techniques for Measuring Fracture Toughness: I, Direct Crack Measurements. J. Am. Ceram. Soc..

[33] (2015). Thermal Spraying-Evaluation of Adhesion/Cohesion of Thermal Sprayed Ceramic Coatings by Transverse Scratch Testing.

[34] (2023). Standard Test Methods for Detecting Detrimental Intermetallic Phase in Duplex Austenitic/Ferritic Stainless Steels.

[35] Ksiazek M., Łyp-Wrońska K. (2024). An HVOF-sprayed (Cr_3_C_2_-NiCr+Co) composite coating on ductile cast iron: Microstructure, mechanical properties, and scratch resistance. Materials.

[36] Hirota K., Mitani K., Yoshinaka M., Yamaguchi O. (2005). Simultaneous synthesis and consolidation of chromium carbides (Cr_3_C_2_, Cr_7_C_3_ and Cr_23_C_6_) by pulsed electric-current pressure sintering. Mater. Sci. Eng. A.

[37] Konopka K., Maj M., Kurzydłowski K.J. (2003). Studies of the effect of metal particles on the fracture toughness of ceramic matrix composites. Mater. Charact..

[38] Bartolome J.F., Beltran J.I., Gutierrez-Gonzalez C.F., Moya J.S., Pecharroman C., Munoz M.C. (2008). Influence of Ceramic–Metal Interface Adhesion on Crack Growth Resistance of ZrO_2_–Nb Ceramic Matrix Composites. Acta Mater..

